# An Investigation on Chinese Public Acceptance of COVID-19 Prevention Measures

**DOI:** 10.3390/ijerph19095087

**Published:** 2022-04-22

**Authors:** Ao Zhang, Hao Yang, Shuning Tong, Jingqi Gao

**Affiliations:** 1 School of Engineering and Technology, China University of Geosciences (Beijing), Beijing 100083, China; 2002200096@cugb.edu.cn (A.Z.); 2002190082@cugb.edu.cn (J.G.); 2 Emergency Management Department of Xinjiang Uygur Autonomous Region, Urumqi 830011, China; tongshuning@163.com

**Keywords:** COVID-19, emergency prevention and control measures, acceptance, epidemic, public acceptance

## Abstract

China has basically succeeded in bringing the COVID-19 epidemic under control, thanks to a timely series of effective prevention and control measures taken by the Chinese government. In this study, a public acceptance questionnaire of epidemic prevention measures was designed to investigate the influencing factors of public acceptance. A total of 2062 samples were collected from 8 March 2020 to 9 April 2020, and Independent-Samples T-Test and One-way ANOVA were used to analyze the data collected in the questionnaire in SPSS version 22.0. The results show that age and educational level have a significant influence on public acceptance. With the development of the epidemic, the acceptability grew generally higher. The public acceptance of traffic measures is the highest. This study summarises China’s scientific experience in the fight against COVID-19 and the differences in public acceptance. It can provide a positive reference for the development of epidemic prevention in other countries.

## 1. Introduction

As we all know, the novel Coronavirus (COVID-19) pandemic is disrupting the world and causing serious damage to human life and economic activities. The World Health Organisation (WHO) has classified it as a “global pandemic public health emergency”. In the face of a major epidemic, the Chinese government has demonstrated its strong disposal, and response capabilities, organizational mobilization, and effective implementation, demonstrating its strength, spirit and efficiency, setting an example and providing “world experience” for other countries in their epidemic prevention work. Currently, China’s epidemic prevention and control situation demonstrates a positive trend. China’s COVID-19 prevention and control strategy has effectively curbed the rapid spread of COVID-19 and protected hundreds of thousands of Chinese people [1,2]. When the epidemic broke out in the Western World, many countries did not implement timely blockade and other measures to contain the spread of the epidemic [3]. The novel coronavirus outbreak has been able to spread to many countries since its inception. In the face of such a serious and highly contagious disease and the complex situation it creates, the measures taken by China are the right ones [4]. In addition, in some hard-hit countries, some officials hold different attitudes toward preventive measures such as wearing masks and maintaining safe social distancing, leading to a low public acceptance of preventive measures [5]. Public acceptance of measures is an important indicator of the effectiveness of the prevention and control measures taken by the government, and the effectiveness of measures directly affects the development trend of the epidemic [6]. Therefore, public acceptance of measures has become one of the most important factors for the effective control of the epidemic [6]. However, there are few pieces of research or literature on public acceptance of measures taken to prevent and control the outbreak. Acceptance refers to the audience’s subjective acceptance of the product or policy, which usually reflects the practicality and effectiveness of the product or policy. Most acceptance studies are conducted in the form of questionnaires to explore the public’s attitude towards products or policies, which can be used to study the public’s views on emergency prevention and control policies during the epidemic [7,8,9].

Existing acceptability studies are divided according to survey objects, generally including public acceptance, consumer acceptance, user acceptance, patient acceptance, etc., with more emphasis on new energy, high-tech, medicine, economic development, education, land development and utilization and other fields, e.g., the acceptance of the renewable energy system by society was investigated through questionnaires, so as to analyze and summarize the obstacles to the application of the renewable energy system and increase consumer acceptance of renewable energy [10,11,12]. Another example is to study the acceptance of genetically modified food in a certain region, so as to analyze the impact of genetically modified food on the local economy [13,14]. In clinical medicine, some studies aim at controversial medical issues such as organ transplantation, to obtain the acceptance of the public represented by patients and patients’ families, so as to improve medical measures and improve medical standards [15]. In general, there are few studies on the acceptance of COVID-19 emergency prevention and control measures, most of which are based on summarizing experience, and finally putting forward prevention and control suggestions for public health emergencies, e.g., Malesza [16] used questionnaires to survey the acceptance and intake of the COVID-19 vaccine among older Germans over 75 years old, suggesting that health authorities should focus on vaccine-related factors rather than aspects related to the illness to improve the vaccination uptake rates during the COVID-19 pandemic; Betsch [17] assessed public acceptance of school-related mask policies of parents and non-parents alongside demographics information, trust in institutions, knowledge about COVID-19 and protective behaviors, as well as risk perceptions and thought that implementing mask policies in school will require intense communication and the acceptance of these policies with regard to teachers and pupils should be considered as well; Zhao [18] believes that China’s experience will be helpful to the rest of the world, and suggests that systematic measures be taken to curb the global spread of COVID-19. In this paper, the public’s acceptance of prevention and control measures is analyzed and studied through a questionnaire survey, and the differences in acceptance among different groups and the changing trend of acceptance are discussed, so as to put forward suggestions and opinions that reflect the public’s wishes.

This study was conducted to examine the acceptance of COVID-19 control measures by personnel in the Chinese region, focusing mainly on management policy instruments. Moreover, there is little international research literature on epidemic measures, and this study contributes to research in the Chinese context by understanding differences in the public acceptance of COVID-19 emergency prevention and control measures and provides an empirical basis for the development of emergency management policies.

## 2. Methods

### 2.1. Procedure

By means of a literature review, this study summarized and optimized the scales of previous acceptance studies. In combination with the emergency prevention and control measures adopted by various regions during the outbreak of the COVID-19 epidemic in China from January to March 2020, a questionnaire was designed to investigate public acceptance of COVID-19 epidemic control measures, and data were obtained. Then, a descriptive statistical analysis, independent-sample t-test and one-way ANOVA were conducted with IBM SPSS Statistics version 22 (IBM SPSS Inc., Chicago, IL, USA) to investigate the differences in acceptance between groups with different demographic characteristics and the trend of acceptance over time.

### 2.2. Questionnaire Design

In this survey, the following variables were used to measure public acceptance based on the existing acceptance studies, while taking into account the characteristics of public health emergencies and the development of epidemics, and finally, they were based on the main epidemic prevention measures, as defined below:

- Epidemic awareness level: the degree of public understanding of the basic information of the epidemic situation, including the characteristics of the virus, the spread of the epidemic situation and other epidemic-related information.
- Measures acceptance: the public’s understanding of the epidemic prevention and control decisions and the frequency of participation, as well as the public’s subjective satisfaction with the decision after understanding the epidemic prevention and control decisions.
- Demographic characteristics: basic personal information such as gender, age, educational level, number of family members, etc.
- Traffic measures effectiveness: the extent and effect of traffic measures on epidemic prevention and control.
- Real economy type measures effectiveness: the extent and effect of real economy type measures on epidemic prevention and control.
- Educational measures effectiveness: the extent and effect of educational measures on epidemic prevention and control.
- Recreational activity measures effectiveness: the extent and effect of recreational activity measures on epidemic prevention and control.
- Other measures effectiveness: the extent and effect of other measures on epidemic prevention and control.

This study adopted the method of a questionnaire survey to conduct an empirical investigation. The questionnaire included the following three parts: questionnaire description, basic personal information and the measurement of public acceptance of COVID-19 emergency prevention and control measures.

In the design of the questionnaire, the basic information of the individual (BI) was obtained using general multiple-choice questions, with a total of six questions, marked as Bi-1, Bi-2… and so on. The public’s acceptance of the novel Coronavirus emergency prevention and control measures was measured using Likert’s five-point method (SC), with 1–5 representing “strongly inconsistent”, “not quite consistent”, “consistent”, “quite consistent”, “strongly consistent”, in ascending order, with a total of 18 questions, marked as Sc-1, Sc-2… and so on. See the Appendix A for a detailed overview of the items in the questionnaire.

### 2.3. Questionnaire Pretest and Distribution

In this study, a small-scale pre-test was conducted before the formal questionnaire. The preliminarily designed questionnaire was distributed to 20 people in the research group, to classmates, relatives and friends on a small scale, and the results were used to judge whether the questions in the questionnaire were reasonable and valid. During the questionnaire pre-test, the team members found some problems. In terms of Likert’s five-point method, the description was not clear, which would easily cause difficulties and bias in the respondents’ understanding, which would directly affect any potential inconsistencies between the data collected by the respondents and the actual situation. Therefore, corresponding modifications were made to the unclear questions. In addition, there were problems of confusion and logical incoherence in the order of questions, so the order of questions was appropriately adjusted and sorted according to the type and content of questions, in preparation for the next reliability analysis of the modified questionnaire.

In this study, Cronbach’s α coefficient was used to measure the reliability of the questionnaire. The reliability of each dimension of the scale was tested, and the values obtained were greater than 0.5, which proved that the reliability of each dimension met the requirements. Finally, after excluding the questions used to collect demographic indicators, the Cronbach’s α coefficient for the remaining 21-item scale was 0.897, which is above 0.7, which means that the questionnaire in this study has a high reliability.

The questionnaire was prepared and distributed on a questionnaire distribution website, and then disseminated and diffused in the form of websites and social media, and the sample was drawn by snowball sampling. The survey population included citizens over 18 years old and under 65 years old who lived in China during the COVID-19 epidemic. All questionnaires were distributed and collected through the web-page version provided by https://www.wjx.cn (accessed on 8 February 2020). Before the questionnaire was distributed, the purpose of the study was explained to research participants. The questionnaire was only used for the academic investigation during the epidemic period to ensure the privacy of the research participants. All patients involved in this study gave their informed consent. Institutional review board approval of our college was obtained for this study.

## 3. Results

### 3.1. Questionnaire Overview

A total of 2101 questionnaires were recovered from 8 March 2020, to 9 April 2020, and 39 questionnaires were excluded as they did not meet the requirements. The number of valid questionnaires was 2062, with an effective recovery rate of about 98.14%. Among the respondents, 48.74% were male, and 51.25% were female. The age ranged between 16 and 60 years old, with 0.53% of participants being adolescents under 18, while 29.39% were young people between 18 and 25, 28.23% were young people between 26 and 30, 24.68% were middle-aged people between 31 and 40, 11.06% were middle-aged people between 41 and 50, 4.95% were middle-aged people between 51 and 60, and 1.16% were older adults over 60 years old. In terms of educational level, 2.91% received junior high school education, 12.51% received senior high school education, 26.24% received junior college education, 50.53% held a bachelor’s degree, and 7.81% held a master’s degree or above.

In addition, the number of respondents from all regions of the country was relatively average, including a large number of people from the worst-hit areas, with 5.09% from Hubei province and 5.00% from Beijing. As for the occupations of the respondents, 21.58% were undergraduates or postgraduates, and 6.55% were engaged in finance and insurance.

The basic demographic statistics of the sample in this study are shown in Table 1, Table 2 and Table 3.

### 3.2. Understanding of the Epidemic and Attitude towards Measures

**Figure 1:**
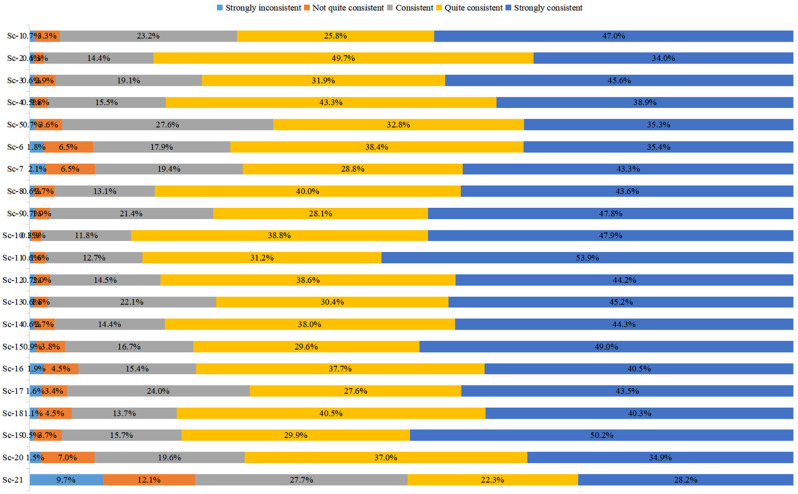
Sample responses on the scale of preventive measures acceptance.

To ensure the accuracy and effectiveness of the research data, the respondents’ understanding of the epidemic situation and the prevention and control measures was collected in the questionnaire, to ensure that the respondents could correctly understand the effective degree of the epidemic prevention measures during the epidemic. Figure 1 shows the sample’s understanding of the epidemic and attitude towards measures.

The following data indicate that most of the respondents in this study have a certain understanding of the nature of COVID-19 itself, a certain understanding of the severity of the virus and the epidemic, and demonstrated a certain level of concern and tracking of the development process of the epidemic, and are also aware of the severity of the epidemic and the difference among the stages of its development.

Additionally, on the attitude to measures, the respondents differed in their judgment of the local government’s disclosure of information on the specific situation of the epidemic and whether the emergency measures adopted were timely, effective and accurate.

### 3.3. Relationship between Demographic Characteristics and Acceptance

First, an independent sample t-test was conducted for the Sc-6 and Sc-7 scores of both men and women. The variance of each factor is uniform among different gender groups, indicating that there is no significant difference, and there is no significant difference in the mean values of the two questions among people of different genders (*p* > 0.05). It can be seen that gender has no significant effect on acceptance.

Second, the samples were divided into the following age groups: under 18 years old, 18~25 years old, 21~25 years old, 26~30 years old, 31~40 years old, 41~50 years old, 51~60 years old and above 60 years old. Firstly, an homogeneity of variance test (Levene) was conducted for each age group, and a *p* > 0.05 was considered significant. It can be seen that the variances of the two questions in different age groups were not homogeneous (*p* < 0.05). Therefore, there are significant differences between the two questions in different age groups (*p* < 0.05). The Kruskal–Wallis method was used to conduct non-parametric test, and the test results showed that Sc-7 had significant differences among different age groups (*p* < 0.05). Through pairwise LDS comparison and non-parametric pairwise comparative analysis, combined with the mean scores of the two questions, mean scores and significance levels were obtained, as shown in Table 2. In general, scores increase with age, and scores of people over 40 were found to be significantly higher than those of other ages. So, age can have a significant impact on how people are accepted.

Third, education level was divided into the following five groups: junior high school and below, high school, junior college, bachelor, master and above. An homogeneity test of variance was conducted for groups with different educational levels. As can be seen, the mean square deviation of Sc-6 is not uniform. Therefore, there is a significant difference between different educational levels (*p* < 0.05). Therefore, the Kruskal–Wallis method was used to conduct non-parametric test, and it can be seen that there are significant differences between the two questions among groups with different educational levels (*p* < 0.05). Through LDS pairwise comparison and a non-parameter pairwise comparative analysis, combined with the score mean, the score mean and significance level were obtained, as shown in Table 3. In general, people with high school education score significantly higher for both questions than people with other educational levels. Therefore, it can be concluded that educational level has a significant impact on people’s acceptance.

Finally, the occupations in the sample mainly include 21 categories, such as finance and insurance, information industry, and students. First, the homogeneity of variance test was conducted for the scores of 21 types of occupations. As can be seen from the table, the variance of the two questions for different occupations is uniform (*p* > 0.05). Then, an independent sample one-way ANOVA was conducted for the ten-factor scores of different occupations, and the results are shown in Table 4. As can be seen from the table, there is no significant difference between the scores of different occupations (*p* > 0.05), indicating that occupations do not have a significant influence on the acceptance of the public.

### 3.4. Analysis of the Changing Trend of Public Acceptance

**Figure 2:**
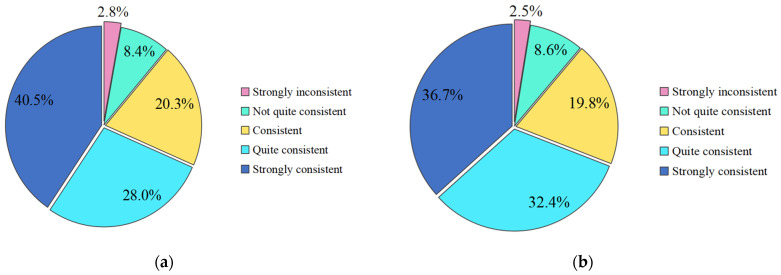
Public acceptance of prevention and control measures in the early phase (8 March 2020 to 23 March 2020). (**a**) Status of Sc-6 responses; (**b**) Status of Sc-7 responses.

**Figure 3:**
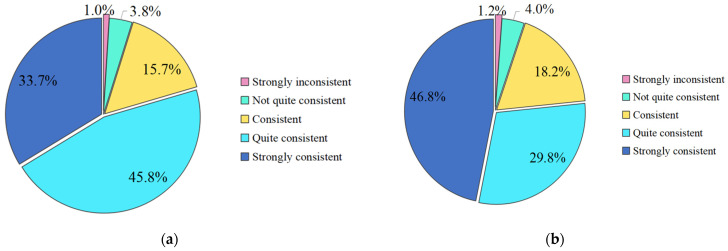
Public acceptance of prevention and control measures in the late phase (23 March 2020 to 9 April 2020). (**a**) Status of Sc-6 responses; (**b**) Status of Sc-7 responses.

In this study, 2062 valid questionnaires were collected from 8 March 2020 to 9 April 2020. The questionnaire data of 1148 questionnaires and 914 questionnaires collected in two time periods of solstice from 8 March 2020 to 23 March 2020, and solstice from 23 March to 9 April 2020, were, respectively, extracted for statistical analysis. Figure 2 and Figure 3, respectively, show the data distribution of sample data collected in Sc-6 and Sc-7 in the first half period. Namely, for the initial phase of the outbreak (later referred to as the early phase) and the second half period, which represents the stable phase of the outbreak (later referred to as the later phase).

The data showed that the average scores of the two questions in the early phase were 3.92 and 3.95, respectively, which meant that the public had a high acceptance of the epidemic control measures. The average scores of the two questions in the late phase were 4.07 and 4.17, respectively, indicating that the public has a very high acceptance of various measures for epidemic prevention and control. During the epidemic, in general, the public was found to have a high acceptance of social isolation, traffic control, screening and testing, dynamic monitoring, diagnosis and treatment, resource allocation, material support and other prevention and control measures. With the development of the epidemic, the trend of the epidemic has been gradually brought under control. China has become more proficient in controlling the epidemic, and the public’s acceptance of the prevention and control measures has increased significantly.

### 3.5. Analysis of Acceptance of Different Epidemic Prevention Measures

Various measures adopted by China have achieved high public acceptance. We generally consider that a mean score of 4.0 or higher for a scale item represents a high level of public acceptance of the measure; conversely, a mean score of 2.0 or lower for the item represents a low level of public acceptance of the measure. A case-by-case analysis of the public acceptance of some types of measures is presented in the following section.

First, this paper analyzes the acceptance of traffic measures by the public, for which the data are shown in Table 5.

Second, this paper analyzes the acceptance of real economy measures by the public, for which the data are shown in Table 6.

Finally, this paper analyzes the acceptance of educational measures by the public, for which the data are shown in Table 7.

## 4. Discussion

### 4.1. Discussion of Results

Since the beginning of the COVID-19 pandemic, public health policymakers have been asked to determine appropriate responses in terms of intensity, duration, and scope [4,19]. With reference to previous public health emergencies, emergency prevention has remained a key component in preventing the worsening of the epidemic and reducing the loss of the outbreak. By comparing the cost of treatment during a large outbreak of hepatitis A with that of prevention and control in advance, it was found that the cost of the latter was much lower than the former [20]. It is important to note that because the questionnaire was released during the peak of the epidemic in China, the findings suggest that the public’s risk perception of COVID-19 outbreak was at a disadvantage and the sentiment towards infectious disease prevention and control was very negative. As a result, during the early stages of the epidemic, the public was generally not receptive to government measures. Theoretical prevention and control measures are prone to bias in practice. Ajay and Bhargavi [21] believe that, although prevention in advance is more advantageous in theory, it is challenging to implement in practice due to the influence of regional social concepts, cultural traditions and other factors, resulting in low prevention efficiency. However, with the development of the epidemic, public acceptance has gradually increased, which proves that the prevention and control measures taken by China are indeed positive and effective.

To prevent and control COVID-19, full cooperation between the government and the public is essential. What worked well in the case of China’s fight against the new crown epidemic was a combination of policies appropriate to China’s policy style of centralized leadership, cadre mobilization, and review of successful experiences [4]. Shwiff, Katie and Aaron [22] show that treating people with infectious diseases can bring broader economic benefits to countries. At the same time, traffic control measures are able to effectively contain the spread of the epidemic; the traffic control and centralized isolation measures in Wuhan have been effective in disease control and can provide a reference for the prevention and control of the epidemic in other countries [4]. Therefore, the implementation of traffic control, centralized isolation and other measures have become an important consideration for the prevention and control of the epidemic.

Traffic measures, as a unique anti-epidemic mode adopted by China in combination with its national nature and political and social norms, can best reflect the measures of complete isolation, which has higher public acceptance than other types of measures. The urban blockade of Wuhan, Hubei province, initially suppressed the spread of the epidemic. With the development of the epidemic, and according to the epidemic situation of other western countries, measures such as traffic control are one of the most effective measures to curb the spread of COVID-19 [4].

The epidemic has had a tremendously negative impact on the country’s economic development. Measures of the real economy are also necessary to minimize the damage and ensure the continuation of the daily life of the public as much as possible. At the public level, the priority is to ensure the daily life of the public during the epidemic, while at the national level, restoring the national economy as soon as possible is advisable. As it is directly related to the daily life of the public, the public acceptance of this part of such measures is also very high. The epidemic has dealt a huge blow to the real economy. As the epidemic has gradually cooled down, China has successively introduced policies and regulations to recover the real economy.

The severity of the epidemic in China comes at a time when school and university students are vacationing and the new semester is starting. Schools are responding to COVID-19 by delaying the start of classes and changing teaching patterns. Both the students themselves and their guardians have a high acceptance of such measures. It can be seen that the mode of postponing the opening of school and changing the teaching mode played a great role during the epidemic. It can not only effectively control the spread of the epidemic, but also does not affect the development of education.

Currently, the epidemic is cooling in China. Colleges all over the country have adopted semi-closed management styles to strictly prevent the outbreak of COVID-19 again. Meanwhile, they have updated their mode of teaching and have adopted a combination of offline and online methods to complete their education work, which has played an effective role in the prevention and control of the epidemic.

Finally, due to the rapid development of the epidemic, other different preventive and control measures, including vaccination, have emerged during the regular phase of the epidemic. The public acceptance of measures at the initial stage of epidemic prevention has to some extent influenced the public acceptance of measures such as vaccination. For example, Francesca Gallè et al. [23] evaluated the acceptance of COVID-19 vaccination in a sample of older adults in southern Italy and found that the acceptance of COVID-19 vaccination was positively associated with a higher education level, among other findings. Future studies should include a consideration of the acceptability of vaccination, precision prevention and control, and other preventive measures to enrich the research in this area in the Chinese context.

### 4.2. Research Contributions

As an investigation and study during the COVID-19 epidemic, this study paid close attention to the development of the epidemic and analyzed the implementation of epidemic control measures from the perspective of the public, which is of profound significance to the prevention and control of the epidemic.

This study focused on one of the most important factors affecting the effective control of the epidemic—the public acceptance of the epidemic prevention measures. The questionnaire was designed to focus on acceptance in order to explore the differences in the public’s acceptance of the epidemic prevention measures adopted in China among different demographic characteristics, and to further explore how to reasonably deal with public health emergency-related prevention and control measures.

Compared with other studies related to the epidemic period, this study explored the effect of gender, age and education level on the public acceptance of epidemic prevention measures in addition to the change in public acceptance over time in 2020. The treatment of the data obtained from the questionnaire in this paper differs from other studies due to the additional sections on changes over time and comparisons of different types of measures. All these data analyses reflect a policy mix comprising traditional measures, i.e., strict community lockdown, cross-jurisdictional mobilization of resources and officials’ sanctions which contributed to the eventual effectiveness of China’s response to the pandemic [4]. In addition, this study can provide a reference and ideas for the development of epidemic prevention guidelines in other socialist countries, and provides some contributions for the international level.

### 4.3. Research Limitations

This study has some limitations in terms of research methods and contents due to the limitations in manpower and time and place, which will be summarized in this section to provide reference for future researchers.

(1) The content of the questionnaire in this study was prepared only based on the existing emergency prevention and control measures in the country before and during the middle of this epidemic, ignoring the recovery measures during the normalization phase of the epidemic, and only common demographic characteristics such as gender, age, and education level were considered when conducting the acceptance difference study, ignoring other demographic characteristic factors. In the future, these factors could be added and the results obtained would be more accurate and detailed.
(2) Due to time, location, and effort constraints, the questionnaire data obtained in this study are regionally concentrated and age-group concentrated, with the sample generally concentrated between the ages of 18 and 30 and the regions concentrated in Liaoning and Beijing. In future studies, the accuracy of the data can be further improved by collecting richer questionnaires in multiple ways and over a longer period of time in order to increase the sample size, improve the diversity of the sample, and average the proportion of the sample distribution.

## 5. Conclusions

After investigating the COVID-19 control work report issued by various provinces and cities of China, this study summarized and collated the corresponding measures taken since the discovery of the epidemic, designed and distributed questionnaires, and the following conclusions were drawn:

1. There was no significant difference in the cognition of measures between different gender and occupational groups; respondents aged between 40 and 60 were more receptive to the measures than respondents of other age groups. Respondents with a bachelor’s degree or above were more receptive to the measures than respondents of other age groups.
2. The public has a high acceptance of emergency prevention and control measures on the whole. With the development of the epidemic, the acceptance increased significantly under the comparison between the government and relevant media and other countries experiencing the severe epidemic.
3. All kinds of measures are highly accepted by the public, among which traffic measures have the highest acceptance.
4. With the improvement of the epidemic situation, public acceptance has gradually increased. Relevant measures can provide reference for other countries during the epidemic.

This paper explores the factors influencing public acceptance of epidemic prevention measures as a reflection of the effectiveness of the measures taken by the Chinese government during the outbreak, enriching the research thinking in the public perspective of related studies. At the same time, future research is required to determine whether the public’s acceptance of epidemic prevention measures will change in the face of the current changes in the epidemic, such as the emergence of new variants of delta and omicron, and whether the government should make changes in response.

## Figures and Tables

**Table 1:** Distribution of the general characteristics of the participants.

Variables	Item	Absolute Frequency	Percentage	Cumulative Percentage
Sex	Male	1005	48.74	
	Female	1057	51.26	
Age	Below 18	11	0.53	0.53
	18–25	606	29.39	29.92
	26–30	582	28.23	58.15
	31–40	509	24.68	82.83
	41–50	228	11.06	93.89
	51–60	102	4.95	98.84
	Above 60	24	1.16	100
Education level	Junior high school and below	60	2.91	2.91
	High school	258	12.51	15.42
	Junior college	541	26.24	41.66
	Bachelor	1042	50.53	92.19
	Master and above	161	7.81	100

**Table 2:** Mean score and significance level of people of different ages.

Item	Below 18 (11)	18–25 (606)	26–30 (582)	31–40 (509)	41–50 (228)	51–60 (102)	Above 60 (24)	*p*
Sc-6	3.55 ± 1.13	3.92 ± 1.02	4.01 ± 0.93	3.99 ± 0.99	4.07 ± 0.93	4.05 ± 1.04	4.42 ± 0.83	0.068
Sc-7	3.27 ± 1.27	3.95 ± 1.01	4.09 ± 1.00	4.02 ± 1.10	4.13 ± 1.04	4.27 ± 1.00	4.48 ± 0.72	0.000 *

* *p* < 0.05.

**Table 3:** Mean score and significance level of people of different degree levels.

Item	Junior High School and Below (60)	High School (258)	Junior College (541)	Bachelor (1042)	Master and Above (161)	*p*
Sc-6	4.15 ± 1.02	4.17 ± 0.90	4.03 ± 0.92	3.94 ± 1.02	3.84 ± 0.99	0.001 *
Sc-7	4.23 ± 0.98	4.26 ± 1.01	4.18 ± 0.92	3.94 ± 1.06	3.89 ± 1.00	0.000 *

* *p* < 0.05.

**Table 4:** One-way ANOVA results of scores of people with different occupations.

**Item**	**Agriculture**	**Mining**	**Manufacturing**	**Water Resources and Hydropower**	**Real Estate**
	**(58)**	**(38)**	**(108)**	**(115)**	**(105)**
Sc-6	4.24 ± 0.73	3.97 ± 0.89	3.99 ± 0.95	4.03 ± 0.85	3.92 ± 1.04
Sc-7	4.34 ± 0.87	3.89 ± 0.92	4.10 ± 0.91	4.01 ± 1.00	3.92 ± 1.15
**Item**	**Modern Logistics**	**Finance/Insurance**	**Information**	**Wholesale/Retail**	**Accommodation/Catering**
	**(120)**	**(135)**	**(128)**	**(93)**	**(97)**
Sc-6	4.08 ± 0.89	4.01 ± 1.01	3.98 ± 0.92	3.92 ± 0.98	3.89 ± 1.06
Sc-7	4.15 ± 1.00	3.99 ± 1.10	4.05 ± 1.07	4.05 ± 0.98	4.04 ± 1.05
**Item**	**Environmental and Public Utilities Management**	**Leasing and Business Services**	**Residential Service**	**Education**	**Recreation and Entertainment**
	**(86)**	**(57)**	**(50)**	**(91)**	**(46)**
Sc-6	3.91 ± 1.00	4.04 ± 0.94	3.70 ± 1.27	4.08 ± 0.96	3.74 ± 0.91
Sc-7	4.09 ± 1.03	3.95 ± 1.09	3.86 ± 1.21	4.09 ± 1.05	3.87 ± 1.24
**Item**	**Medicine and Health**	**Government Departments and Social Organizations**	**Army/Police**	**Freelancer**	**Retired and Housewife**
	**(80)**	**(51)**	**(23)**	**(74)**	**(62)**
Sc-6	4.08 ± 0.93	4.06 ± 0.99	4.04 ± 1.11	3.70 ± 1.11	4.10 ± 1.00
Sc-7	4.23 ± 1.06	4.12 ± 1.09	3.74 ± 1.01	3.96 ± 1.16	4.18 ± 0.97
**Item**	**College or Graduate Students**			***p***	
	**(445)**				
Sc-6	4.02 ± 1.00			0.009 *	
Sc-7	4.04 ± 0.99			0.371	

* *p* < 0.05.

**Table 5:** Acceptance of traffic measures.

Question Number	Strongly Inconsistent	Not Quite Consistent	Consistent	Quite Consistent	Strongly Consistent	M
Sc-11	0.58%	1.55%	12.71%	31.23%	53.93%	4.36
Sc-12	0.68%	2.04%	14.45%	38.60%	44.23%	4.24
Sc-15	0.87%	3.83%	16.73%	29.58%	48.98%	4.22
M	0.71%	2.47%	14.63%	33.14%	49.05%	4.27

Notes: M is for mean.

**Table 6:** Acceptance of real economy measures.

Question Number	Strongly Inconsistent	Not Quite Consistent	Consistent	Quite Consistent	Strongly Consistent	M
Sc-14	0.63%	2.67%	14.40%	37.97%	44.33%	4.23
Sc-16	1.89%	4.51%	15.42%	37.73%	40.45%	4.10
M	1.26%	3.59%	14.91%	37.85%	42.39%	4.17

Notes: M is for mean.

**Table 7:** Acceptance of educational measures.

Question Number	Strongly Inconsistent	Not Quite Consistent	Consistent	Quite Consistent	Strongly Consistent	M
Sc-19	0.53%	3.69%	15.71%	29.87%	50.19%	4.26
Sc-20	1.50%	7.03%	19.64%	36.95%	34.87%	3.97
M	1.02%	5.36%	17.68%	33.41%	42.53%	4.12

Notes: M is for mean.

## Data Availability

The data presented in this study are available in the Web of Science core database.

## Appendix A

**Table A1:** Public acceptance of COVID-19 preparedness measures questionnaire.

Research Variables	Question Number	Question of Measurement
Demographic characteristics	Bi-1	Gender?
	Bi-2	Age?
	Bi-3	Level of education (including ongoing)?
	Bi-4	Place of residence?
	Bi-5	Number of family members (including yourself)?
	Bi-6	Occupational area?
Epidemic awareness level	Sc-1	I know a lot about the symptoms of COVID-19.
	Sc-2	I know a lot about how COVID-19 is transmitted.
	Sc-3	I know a lot about prevention measures for COVID-19.
	Sc-4	I know a lot about cases of COVID-19 infection.
Measures acceptance	Sc-5	I know a lot about the emergency measures taken by my local government.
	Sc-6	I believe that my local government is timely and accurate about the specific situation of the epidemic.
	Sc-7	I think it is timely for my local government to take emergency prevention and control measures.
Traffic measures effectiveness	Sc-11	I believe that city closures are important to the prevention and control of the epidemic.
	Sc-12	I believe that the closed management of the community plays an important role in the prevention and control of the epidemic.
	Sc-15	I believe that traffic control measures (such as banning the passage of motor vehicles) play an important role in the prevention and control of the epidemic.
Real economy measures effectiveness	Sc-14	I believe that delayed resumption of work and post-resumption of protection (health monitoring, etc.) play an important role in the prevention and control of the epidemic.
	Sc-16	I believe that controlling the price increase of epidemic prevention products and basic daily necessities plays an important role in epidemic prevention and control.
Educational measures effectiveness	Sc-19	I believe that the postponement of school opening has played an important role in the prevention and control of the epidemic.
	Sc-20	I think the adoption of online teaching in schools has played an important role in the epidemic.
Recreational activity measures effectiveness	Sc-13	I believe that limiting or stopping crowd gathering will play an important role in epidemic prevention and control.
	Sc-21	I believe that reducing entertainment programs will play an important role in the prevention and control of the epidemic.
Other measures effectiveness	Sc-8	I believe that publicly confirming the trajectory of patients is important for epidemic prevention and control.
	Sc-9	I believe that disclosure of personal protective measures is important for epidemic prevention and control.
	Sc-10	I think the construction of special hospitals (Raytheon hospital, Vulcan Hospital, etc.) will play an important role in the prevention and control of the epidemic.
	Sc-17	I believe that the punishment of concealment, delay and false reporting of the epidemic situation will play an important role in the prevention and control of the epidemic.
	Sc-18	I believe that strengthening the punishment for spreading rumors during the epidemic will play an important role in the prevention and control of the epidemic.
